# Selected Properties of the Surface Layer of C45 Steel Samples after Slide Burnishing

**DOI:** 10.3390/ma16196513

**Published:** 2023-09-30

**Authors:** Agnieszka Skoczylas, Mariusz Kłonica

**Affiliations:** Department of Production Engineering, Mechanical Engineering Faculty, Lublin University of Technology, 20-618 Lublin, Poland; m.klonica@pollub.pl

**Keywords:** slide burnishing, surface roughness, microhardness, machining fluid, surface free energy

## Abstract

This paper presents the experimental results of a study investigating the impact of the machining fluid type, the variable factor, used in slide burnishing on 2D and 3D surface roughness; surface topography; Abbott–Firestone curve shape; microhardness; and SFE (surface free energy). In the experiment, pre-ground, ringed samples of C45 steel were used. The results showed an over eight-fold decrease in the value of the Ra (arithmetical mean deviation) parameter and over a five-fold decrease in the Rt (total height of profile) parameter in relation to their values after grinding. The parameters Rpk (reduced peak height), Rk (core roughness depth), and Rvk (reduced valley depth) were also reduced. The Abbott–Firestone curve after slide burnishing changed its angle of inclination (it was more flattened), and the material ratio Smr increased. The reduction in the Rpk and Rk parameters and increased material ratio will most likely contribute to restoring the functionality of these surfaces (increased resistance to abrasive wear). After slide burnishing, the maximum 25% increase in microhardness was obtained compared to the value after grinding, while the layer thickness was 20 μm. The surface energy of elements subjected to slide burnishing using various machining fluids slightly increased, or its value was close to that of the ground surface. The most favourable properties of the surface layer in terms of mating between two elements were obtained for a part that was slide-burnished with a mixture of oil + polymethyl methacrylate (PMM) + molybdenum disulphide (MoS_2_).

## 1. Introduction

Machinery components shaped by machining very often require finishing. Currently, ball burnishing [1,2], slide burnishing [3], shot peening [4,5], and brushing [6,7] are successfully used as finishing treatments.

Ball burnishing causes a reductiontheroughness parameters of a part [8], which contributes to its improved corrosion resistance [2]. This is due to the fact that compressive residual stresses are formed in the surface layer, and the microhardness of this layer increases [9,10]. Ball burnishing also leads to increased fatigue strength [11,12], higher resistance to fretting fatigue, and reduced wear volume [13].

The use of shot peening causes favourable changes in the physical properties of the surface layer, which, in turn, improves the functional properties of a part. In the surface layer of a shot-peened part, compressive residual stresses are induced [14], which improves fatigue life of machine components [15]. The balls acting on the surface of the workpiece during shot peening cause microstructural changes and grain fragmentation [16], which results in increased microhardness [17], higher corrosion resistance and wear performance [18], as well as improved strength [19]. As a result of shot peening, however, defects are formed in the surface layer [20,21], and they can be assessed using annihilation techniques [22] and recurrence and entropy methods [23]. Shot peening also results in reduced surface roughness [24] and modified surface topography [25]. Centrifugal shot peening [26], by applying the microhammering head, can be used formodifications effects after electro-discharge machining (EDM) [27]. Electrodes, used in EDM, may be produced via Fused Deposition Modeling (FDM) [28] using ABS P400 polymer material [29] or sintered material [30].

Currently, forshot peening, a laser beam (laser shot peening) and a water jet (water jet peening or cavitation peening) are successfully used as shot elements [31,32]. The use of laser shot peening (LSP) hasmany benefits over conventional shot peening. These are better surface finish, higher depths of residual stress, and uniform distribution of intensity [33]. After laser shot peening, fatigue strength increases [34], both for additively and classically manufactured elements [35]. It is possible to effectively promote surface grain refinement and induce a deep compressive residual stress field in Inconel 718 samples [36].

Nowadays, laser shot peening and conventional shot peening are often combined in one operation [34,37]. Brushing is successfully used to remove both burrs from the edges of elements after the milling process [38] and surface defects [39], as well as to change the geometric structure of the surface after an abrasive waterjet [40].

Slide burnishing is classified as a static type of burnishing. In slide burnishing, the burnishing element pressed with force, usually in the range of 20 ÷ 250 N [41,42,43], is in constant contact with the surface of the workpiece. There is sliding friction between the surface of the workpiece and the surface of the burnishing element (tip). The tip of the slide burnishing tool can be in the shape of a torus, cylinder, or spherical cap with a radius of 1 ÷ 4 mm. Slide burnishing allows for machining low-stiffness elements (e.g., thin-walled sleeves) [3], parts for pre-milling [44,45], small elements, hardened steels [46], objects with holes [47], and objects with galvanic-diffusion coatings [48]. Slide burnishing is widely used in the aerospace and automotive, among other industries [3]. The use of slide burnishing changes the stereometric and physical properties of the surface layer.

Slide burnishing is primarily used as a smoothing treatment. The main purpose of this type of burnishing is to reduce surface roughness. The slide burnishing of magnesium alloy elements makes it possible to obtain a surface roughness of Sa < 0.4 μm [41], while for D16T aircraft aluminium alloy it is possible to obtain Ra = 0.05 μm [49], and for carbon steel Ra = 0.31–0.53 μm [50]. Zielecki et al. [51] used slide burnishing and obtained a reduction in the surface roughness of X19NiCrMo4 steel from 64.1% to 85.8% compared to the value after turning. A study [50] found there was a limit to the smoothing effect and that it depended on the burnishing force and ball diameter.

Slide burnishing also brings positive changes in the physical properties of the surface layer. After slide burnishing of X19NiCrMo4 steel, the microhardness increased by 32% at a depth of 0.018 mm [51], and for chromium–nickel steel AISI 316Ti the increase exceeded 32% [52], while in a study [22] the maximum degree of strengthening was e = 42.74%, and the maximum thickness of the strengthening layer was up to 100 µm. The microhardness of the surface layer after slide burnishing depends on the burnishing speed, feed rate, burnishing force, and the burnishing tip radius [51,52]. In slide burnishing of 17-4 HP stainless steel under the minimum quantity lubrication (MQC) conditions, the maximum surface hardness was obtained by using low burnishing feed and speed yet high burnishing force [53]. Toboła et al. [54] found that for Ti6Al4V titanium alloy, the application of slide burnishing and low-temperature gas nitriding increased the surface hardness by 5–10% without compromising the strength of the core material. In the surface layer of slide-burnished parts, compressive residual stresses are formed [22,51,53]. The residual stresses induced in the surface layer depend on the tool radius and burnishing force [49], and they are very often moreparallel to the burnishing direction rather than in the perpendicular direction (46–51% higher) [51]. The compressive residual stresses retard the formationof fatigue macro-cracks and thus increase the lifetime of slide-burnished parts [52]. As a result of the favourable properties of the surface layer of slide-burnished parts, there is increased fatigue strength [51,52], improved corrosion resistance [55], and abrasion resistance [56]. Slide burnishing also works well for finishing materials that cannot be subjected to heat treatment [57]. It was also found that the geometrical characteristics of surface texture are significant under boundary lubrication friction, while the physical and mechanical characteristics of the surface layer are important under dry friction conditions.

The role of a cutting fluid in the manufacturing process is to cool, lubricate, and reduce friction. These roles are performed by cutting fluids mainly in machining and plastic working. In machining, air cooling is successfully used [58] as a cooling liquid. In the case of shot peening, the main task of machining fluids is to reduce friction in the contact zone between the balls and the machined surface [59]. Due to the impact of the balls on the surface of the workpiece during shot peening, the material moves toward the edge of the resulting impression. The use of a lubricant reduces the friction between the balls and the material, which leads to an increase in the plastic deformation of the material. In shot peening, oils (e.g., transformer oil) or kerosene are used as liquids. The use of a cutting fluid in shot peening increases fatigue strength and has a positive effect on the properties of the surface layer [59]. In order to improve the effectiveness of liquids used in burnishing or shot peening, a surface-active substance is introduced into the liquid [59]. Surface-active substances include EP (Extreme Pressure) additives and polar additives [60]. The addition of EP-type additives to liquids results in the formation of a chemical compound with the treated metal, which acts as a durable lubricating film [59]. Polar additives act on the burnished material as a result of adsorption on the surface of cutting liquid particles. This facilitates plastic deformation of the material and, consequently, reduces its strength. The Rebinder effect is responsible for changes in material properties as a result of surface-active interaction of liquids [61,62]. A distinction is made between the external and the internal nature of the Rebinder effect.

The energy state (SFE—surface free energy) of the surface layer of modern construction materials used in the engineering industry is particularly important in technologies where adhesion is crucial for structural safety. These technologies include adhesives and adhesion, airtight sealing of responsible structures, coating, printing, and sintering techniques [63,64,65,66,67]. For efficient joining and gas welding [68,69] of construction materials, it is necessary to ensure correct preparation of the surface layer of bonded elements and filler materials in the welding process [68,69] and to select adhesives and sealants with optimal properties.

In industrial practice and the world of science, methods for determining surface free energy (SFE) and new ones are being developed. For liquids, these are direct methods, whereas in the case of solids—indirect methods that are primarily based on measuring the contact angle of wetting with measuring liquids (e.g., distilled water and diiodomethane). The most important methods for determining surface free energy include, for example, the Owens–Wendt method, the Chaudhury and Good method, the Neumann method, and others [63,66,67,70,71].

The use of slide burnishing for finishing brings very favourable results, as confirmed by previous studies. An interesting aspect of this problem is to use surface-active additives to oil and then use this type of mixture as a cutting fluid in slide burnishing [72]. Studies [73,74] showed that the free surface energy of samples crushed in different environments was greater than that of ground samples and that the presence of a surface-active additive significantly affected the reduction adhesive wear. The presence of a surface-active additive was also found to affect the dominant wear mode and the wettability of the surface [74]. A previous study [75] demonstrated that the surface roughness parameters associated with the Abbott–Firestone curve could be used to evaluate the functional properties of the workpiece. Therefore, studies were undertaken on slide burnishing using various cutting fluids. The aim of this study was to assess the effect of the type of cutting fluid on the surface topography, surface roughness, surface layer microhardness, and surface free energy (SFE). There are no works showing the influence of the cutting fluid used in slide burnishing on various properties of the surface layer. It seems that the knowledge of all these properties can help select the most advantageous cutting liquid for slide burnishing of C45 steel.

## 2. Materials and Methods

Thin-walled samples with an outer diameter of d = 56 mm, inner diameter of d_o_ = 50 H7, and width of b = 10 mm were used in this study. The ringed samples were made of C45 non-alloy steel. C45 is widely used in the engineering industry. It is used for medium-duty elements of machines and devices, such as shafts, axles, discs, levers, and non-hardened gears. The chemical composition of C45 steel, defined on the basis of the material card, was as follows: C—0.48%, Mn—0.74%, Si—0.36%, P—0.011%; S—0.01%, Cr—0.09%, Ni—0.02%, Mo—0.002%, Fe—rest, and the selected properties of C45 steel, Re = 430 MPa, Rm = 740 MPa, and hardness (min)—250 HB.

Before slide burnishing, the ringed samples were pre-ground. The grinding process was conducted with a grinding speed of *v_s_* = 35 m/s and a grinding depth of a_p_ = 0.01 mm using a cylindrical grinder (aloxite abrasive wheel).

**Figure 1:**
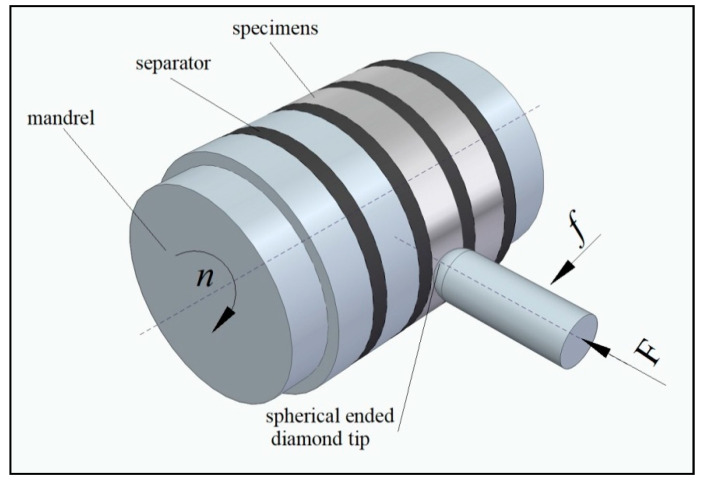
Schematic of slide-burnishing process for ring samples with a burnishing tip made of synthetic diamond.

For the slide burnishing of ringed samples to be carried out on a universal lathe (C11/MB machine made in Bulgaria), the samples were mounted on a mandrel, which was rotated with a speed of *n*. The burnishing tool consisted of a spherical tip with a radius of R = 2.0 mm, and a spring mechanism was pressed against the surface of the samples with a force *F*. The burnishing tool performed a feed motion *f*. Figure 1 shows the schematic of a slide burnishing process for ringed samples made of C45 steel.

The variable in the slide burnishing process was the type of machining liquid. Previous studies on the slide burnishing of titanium alloy Ti6Al2Mo2Cr [72] showed that the type of cutting fluid could have a significant impact on obtained results. The parameters of slide burnishing selected on the basis of previous studies are listed in Table 1.

The 2D surface roughness and 3D topography were measured using the T800 RC120-400 device from Hommel-Etamic, Jenooptik (Jena, Villingen-Schwenningen, Germany). The measuring length was 0.08, while the sampling length was 4.8 mm. Surface roughness parameters were measured in compliance with the EN ISO 25178-2: 2022-06 standard [76]. The following surface roughness parameters were selected for analysis due to their widespread use and the possibility of surface functional characteristics assessment:

Ra—arithmetical mean deviation;

Rt—total height of profile;

Rpk—reduced peak height;

Rk—core roughness depth;

Rvk—reduced valley depth.

The area of the scanned surface was 3 × 3 mm. For the scanned surface area, the material bearing curve (Abbott–Firestone curve) and the material ratio Smr at the cut-off levels of 40% and 60% of the maximum surface height (Sz parameter) were determined.

**Figure 2:**
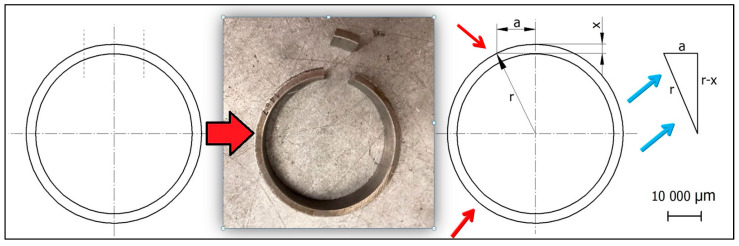
Methodology of the angled micro-section used for microhardness measurements (where: r—radius of the ringed sample, a—distance from the edge of the angled micro-section, x—distance from the edge of the perpendicular micro-section).

The microhardness of the surface and micro-sections was measured using the Leco LM 700at (Leco, St. Joseph, MI, USA) microhardness tester. The Vickers method was employed, assuming the indenter weight of 50 g (HV 0.05). The samples were subjected to standard processing. Measurements were made on angled micro-sections, which made it possible to measure right next to the machined surface. The method of making an angled micro-section and the algorithm of establishing a mathematical dependence are shown in Figure 2. A simple mathematical relationship (Formula (1)) made it possible to calculate the distance to be moved from the edge of the angled micro-section—a, in order to be able to measure the microhardness at a real distance from the edge of the sample—x.

Using the Pythagorean theorem, the following equation was obtained:

(1) $$a^{2}+{\left(r-x\right)}^{2}=r^{2}\;a^{2}+r^{2}-2rx+x^{2}=r^{2}\;\mathrm{assuming}\;\mathrm{that}\;x^{2}\approx 0\;a^{2}=2rx\;a=\sqrt{2rx}$$

where:

*r*—radius of the ringed sample;
*x*—distance from the edge of the perpendicular micro-section;
*a*—distance from the edge of the angled micro-section.

In order to determine surface free energy (SFE), the contact angle was measured. The measurements of the contact angle were carried out using distilled water and diiodomethane. The measuring liquid with a constant density of 4µLwas placed mechanically on a tested surface using a PGX goniometer mechanism, manufactured by FIBRO System AB, Sweden.

In qualitative terms, surface free energy is the amount of work required to create a new unit of area during the division of two balanced phases in a reversible isothermal process. The relation between surface free energy and surface tension is (2):

(2) $$\sigma =\gamma +S\frac{\partial \gamma }{\partial S},$$

where:

*σ*—surface tension;
*γ*—surface free energy;
*S*—unit of area of a given body.

For liquids, the following relationship was assumed (3):

(3) $$\frac{\partial \gamma }{\partial S}=0\;\Rightarrow \sigma =\gamma ,\;\mathrm{for}\;S\neq 0.$$

**Figure 3:**
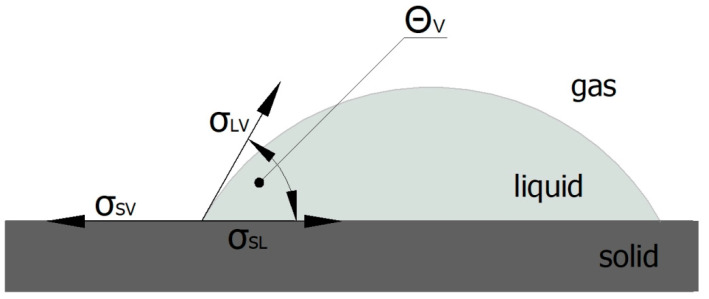
Schematic of Young’s equation and contact angle measurement.

The nature of interactions in a solid–liquid–gas system is described by the characteristic Young’s Equation (4) [67,70], whose graphical interpretation and the way of measuring the contact angle are presented in Figure 3:

(4) $$\sigma_{SV}=\sigma_{SL}+\sigma_{LV}\cos\Theta_{V},$$

where:

*σ_SV_*—surface tension in the solid-gas interface;
*σ_SL_*—surface tension in the solid-liquid interface;
*σ_LV_*—surface tension in the liquid-gas interface;
*Θ_V_*—equilibrium contact angle.

Many methods thatinvolve measuring the contact angle of measuring liquids are based on this equation.

**Figure 4:**
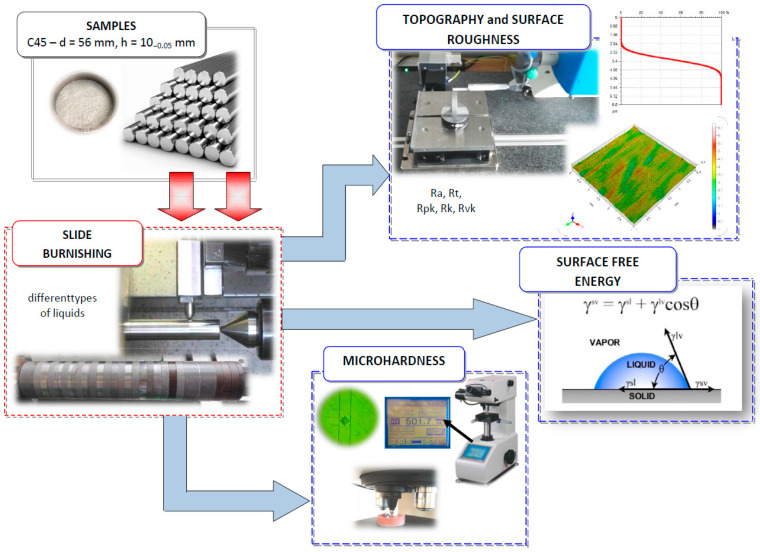
Research methodology (stands, procedures) used in slide burnishing.

Figure 4 shows the test stands and procedures that were used to determine the properties of slide-burnished C45 steel samples with the use of various machining liquids.

## 3. Results and Discussion

### 3.1. Surface Topography and Material Bearing Curve

Table 2 shows the surface topography of C45 steel samples after grinding and slide burnishing using different types of machining liquids. The surface after grinding is characterized by a unidirectional pattern of micro-irregularities, which is the effect of grinding wheel work. Sharp peaks of elevations and numerous depressions are observed in the surface topography. Elevations dominate in the total height of the surface micro-irregularities, which is confirmed by a higher value of the Sp parameter. This shape of the micro-irregularities on the surface is a result of the impact of abrasive grains, leading to grooving and micro-cut occurrence on the surface of the sample. After slide burnishing, the surface topography is changed. As a result of the constant contact of the diamond tip with the surface of the sample, the micro-irregularities formed after grinding are plastically deformed. Their shape is more irregular, which may result from friction and adhesive interaction between the tip and the slide-burnished surface. This phenomenon can be observed in slide burnishing conducted with the following cutting fluids: oil+PMM+MoS_2_, oil+PMM, and oil+MoS_2_. Slide burnishing conducted with a liquid enriched with surface-active additives causes “softening” of the surface, which makes the surface more susceptible to deformation. After slide burnishing with oil and emulsion, the pattern of micro-irregularities is unidirectional and similar to that after grinding, with noticeable elevations and depressions. The 3D amplitude and height parameters after slide burnishing are for all cases lower than their values obtained after grinding (Sa is lowered from 74% to 80%, while Sz from 55% to 73%). It should be noted that there are no large differences in the obtained values of roughness parameters depending on the slide-burnishing medium. The minimum Sa and Sz values were obtained in slide burnishing with oil. In addition to the low values of the Sa, St, Sp, and Sv parameters, the shape parameters are also important, namely—the negative skewness Ssk and the kurtosis value Sku greater than three. Taking into account these criteria, it seems that the best properties in terms of abrasive wear resistance were obtained from slide burnishing with oil+PMM+MoS_2_ and oil+PMM. The values of the coefficients Ssk < 0 and Sku > 3 will probably contribute to the improvement of contact conditions of the friction pair elements by reducing their plasticity indexes and accelerating volume wear reduction. Low Ssk and high Sku surfaces can be “traps” to capture wear particles. The probable changes in the tribological properties of C45 steel elements after sliding burnishing are consistent with the results reported in [56,77]. The obtained absolute values of the Ssk coefficient are smaller than after slide burnishing austenitic stainless steel 317Ti [78].

**Figure 5:**
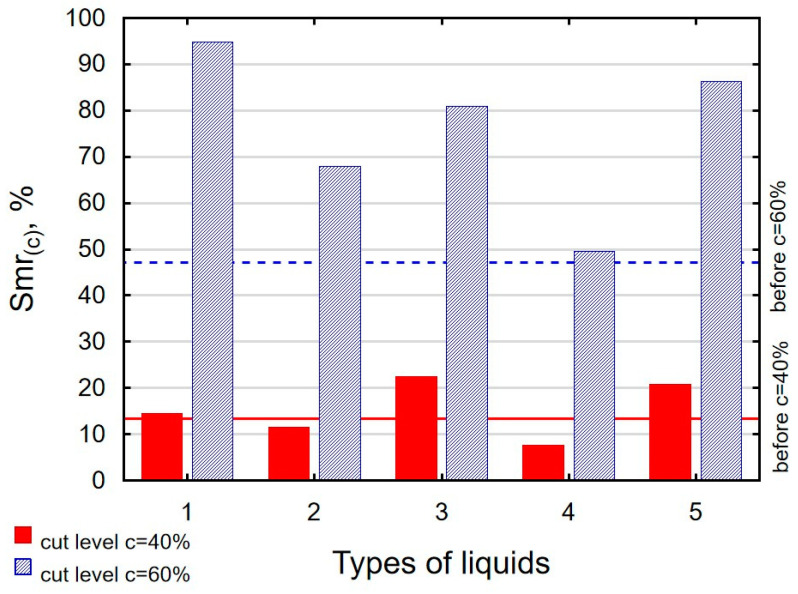
Influence of slide burnishing conducted in various media on the Smr material bearing of C45 steel samples where: 1—slide burnishing in oil+PMM+MoS_2_, 2—slide burnishing in oil+PMM, 3—slide burnishing in oil+MoS_2_, 4—slide burnishing in oil, and 5—slide burnishing in EcoEm-1 emulsion.

In terms of mating, the shape of the material bearing curve and the material ratio Smr are important indicators. Table 3 and Figure 5 present the analysed indicators. The Abbott–Firestone curve after grinding has a degressive–progressive pattern. It is characterized by a large inclination angle. The shape of the curve suggests that we are dealing with a surface with sharp peaks of micro-irregularities, which is confirmed by the topography of the surface after grinding (Table 2). After slide burnishing, the shape of the Abbott–Firestone curve does not change, but its angle of inclination does. The curve is flattened, which may indicate an increase in the resistance to abrasive wear. The most favourable shape of the Abbott–Firestone curve and the highest values of the Smr material ratio were obtained after slide burnishing using oil+PMM+MoS_2_. For the cut-off levels c = 40% and c = 60% for the sample burnished with oil, the Smr value is lower than after the pre-treatment. The result may indicate that a lack of surface-active additives in the machining liquid reduces the “smoothing” effect. These results regarding surface topography confirm the findings reported by the studies presented earlier in this section.

### 3.2. 2D Surface Roughness

**Figure 6:**
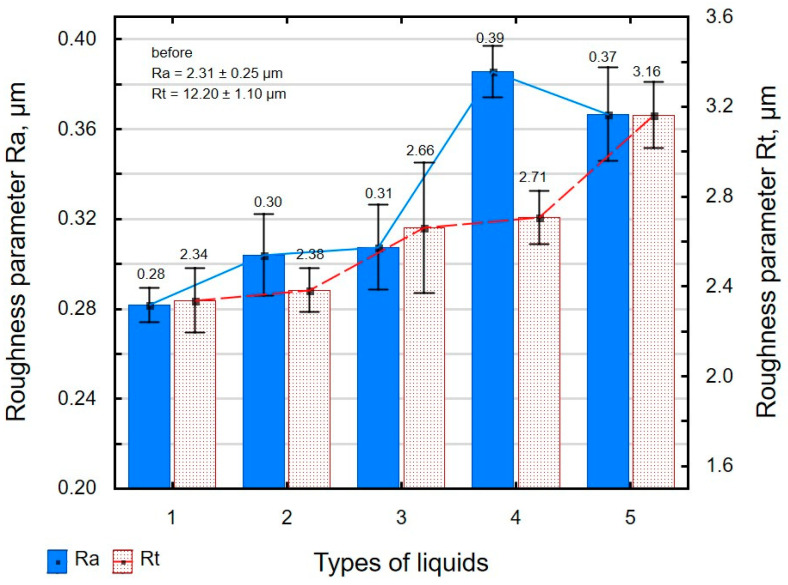
Influence of slide burnishing conducted in various media on the Ra and Rt surface roughness parameters of C45 steel samples where: 1—slide burnishing in oil+PMM+MoS_2_, 2—slide burnishing in oil+PMM, 3—slide burnishing in oil+MoS_2_, 4—slide burnishing in oil, and 5—slide burnishing in EcoEm-1 emulsion.

**Figure 7:**
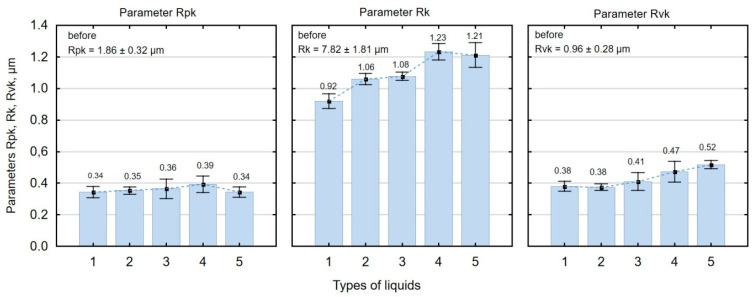
Influence of slide burnishing conducted in various media on the Abbott–Firestone curve parameters of C45 steel samples, where: 1—slide burnishing in oil+PMM+MoS_2_, 2—slide burnishing in oil+PMM, 3—slide burnishing in oil+MoS_2_, 4—slide burnishing in oil, and 5—slide burnishing in EcoEm-1 emulsion.

The influence of the type of cutting fluid on the surface roughness parameters is shown in Figure 6 and Figure 7. Slide burnishing results in a maximum reduction of more than eight times in the Ra parameter and more than five times in the Rt parameter. The greatest changes in surface quality were obtained after slide burnishing using oil with the addition of PMM and MoS_2_. It should be noted that the changes in the surface roughness parameters Ra and Rt as a function of the cutting fluid type do not differ significantly, as the standard deviation bars overlap. A reduction in the surface roughness parameters may be beneficial in terms of tribological properties and fatigue life. The obtained values of surface roughness parameters are higher than those after slide burnishing of X6CrNiTi18 stainless steel (Sa = 0.147 µm, for F = 160N and f = 0.06 mm/rev.) [22] yet lower than after slide burnishing of titanium alloy Ti6Al2Mo2Cr [72] and carbon steel [50].

Slide burnishing also leads to changes in the parameters of the Abbott–Firestone curve (Figure 7). As a result of slide burnishing, the reduced peak height Rpk is decreased from 79% to 82%, and the depth of the surface roughness core Rk from 84% to 88%. The decrease in the Rpk parameter relative to the value after grinding is beneficial due to the wear after the lapping period of the mating parts. On the other hand, the low value of the Rk parameter allows us to conclude that there is a high load capacity of the surface after slide burnishing. As a result of surface smoothing, the Rvk parameter is decreased (reduction from 45% to 60%). This is an undesirable phenomenon due to a decrease in the lubricant retention capacity of the friction pair elements. The changes in the material bearing curve parameters are greater than those observed after slide burnishing of titanium alloy Ti6Al2Mo2Cr [72]. The lowest value of the analysed parameters Rpk, Rk, and Rvk was obtained using oil+PMM+MoS_2_ as a machining medium. In tribological terms, this result is the same as the results of3D shape parameters (Ssk < 0 and Sku > 3).

### 3.3. Microhardness

**Figure 8:**
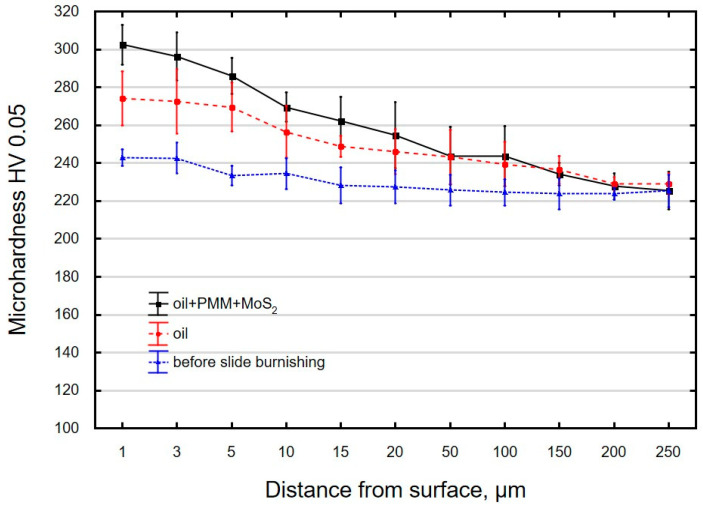
Microhardness distribution the surface layer of the samples afterpre-treatment (grinding) and slide burnishing using oil and oil+PMM+MoS_2_ as liquid.

The use of grinding and slide burnishing causes changes in the microhardness of the surface layer (Figure 8). For the ground sample, the microhardness of the surface layer at a depth of 1 μm is about 9% higher than the microhardness of the core. The thickness of the layer strengthened by grinding is 5 μm. A comparison of the microhardness distribution of samples burnished using different media reveals that the highest microhardness occurs near the surface, at a depth of 1 μm. The maximum microhardness for ground and burnished samples occurs in the region of the most deformed crystals. The microhardness of the samples burnished in oil+PMM+MoS_2_ medium was about 25% higher compared to the ground sample. For the samples burnished in oil, the increase in microhardness was 13%. Based on the distribution of microhardness, it can be concluded that the surface-active additive not only causes an increase in microhardness, but also affects the depth of changes. The use of oil as a cutting fluid causes changes in the microhardness to a depth of approx. 10 μm, while the addition of PMM and MoS_2_ results in an almost two-fold increase in the thickness of the strengthened layer. The obtained increase in microhardness and the depth of changes occurring after treatment are smaller than after slide burnishing of 41Cr4 steel [79].

**Figure 9:**
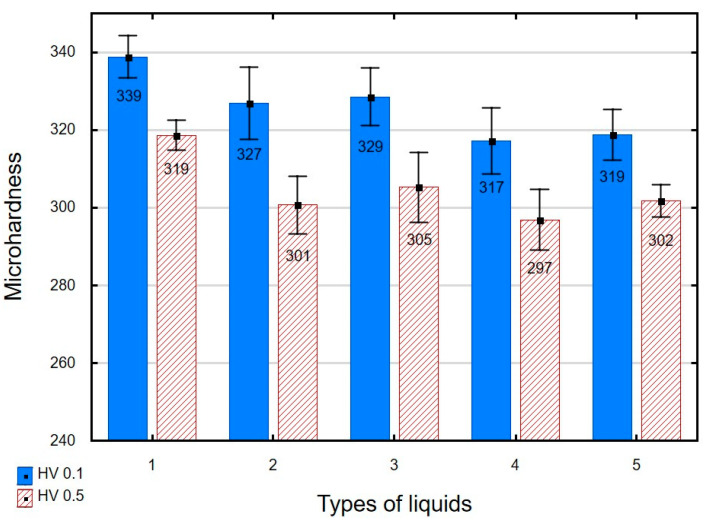
Influence of slide burnishing conducted in various media on the microhardness of C45 steel samples, where: 1—slide burnishing in oil+PMM+MoS_2_, 2—slide burnishing in oil+PMM, 3—slide burnishing in oil+MoS_2_, 4—slide burnishing in oil, and 5—slide burnishing in EcoEm-1 emulsion.

Figure 9 compares the microhardness of samples burnished in different media. The highest microhardness values of HV 0.5 and HV 0.1 were obtained for the samples burnished in oil+PMM+MoS_2_. The presence of a surface-active additive in oil increases the rate of dislocation formation and displacement, thus leading to increased microhardness. The largest increase in microhardness was obtained for the surfaces that were slide burnished using “rich” mixtures (oil+PMM+MoS_2_; oil+PMM; oil+MoS_2_). The smallest changes in surface microhardness were obtained after slide burnishing conducted using emulsion as a cutting fluid. The obtained microhardness values of HV0.5 and HV 0.1 confirm that the presence of a surface-active additive “facilitates” plastic deformation and destruction of material cohesion. The microhardness increase is lower than that obtained after slide burnishing of X19NiCrMo4 steel [51] and X6CrNiTi18 stainless steel [22].

### 3.4. Surface Free Energy (SFE) and Drop Shape

**Figure 10:**
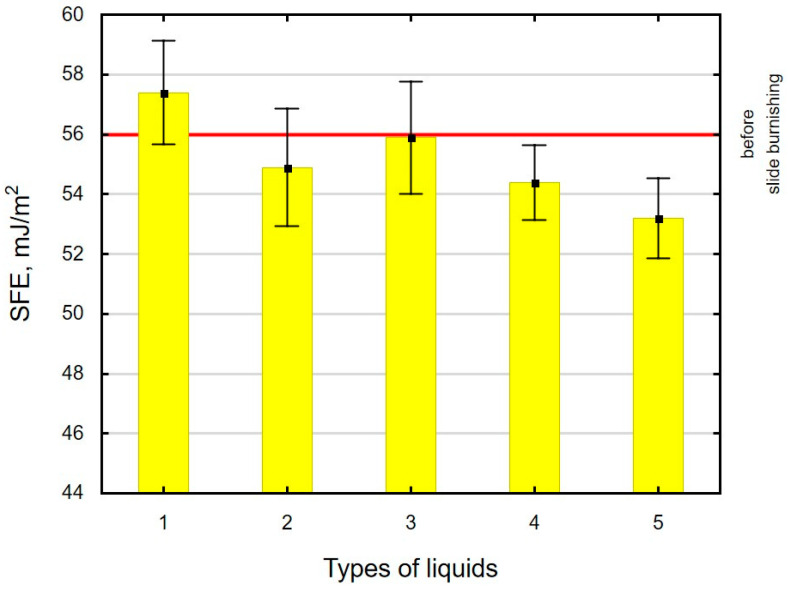
Influence of slide burnishing conducted in various media on the surface free energy of C45 steel samples, where: 1—slide burnishing in oil+PMM+MoS_2_, 2—slide burnishing in oil+PMM, 3—slide burnishing in oil+MoS_2_, 4—slide burnishing in oil, and 5—slide burnishing in EcoEm-1 emulsion.

The experimental results showed that the interaction of the cutting fluid with the surface-active additives led to changes in the SFE. The presence of the surface-active additives caused the SFE to either decrease or retain a value similar to that after grinding (Figure 10). These results differ from those reported in [73,74]. The SFE reduction is conducive to the formation and movement of dislocations in the surface layer, which leads to “easier” deformation of the surface layer (the so-called Rebinder effect) [61]. For oil+PMM+MoS_2_, a slight increase in the SFE was observed in relation to its value after grinding. The results of SFE and surface microhardness demonstrate that for surfaces slide burnished in the medium enriched with surface-active additives, the greatest changes in the tested properties (microhardness, SFE) were obtained, which confirms the occurrence of the Rebinder effect [59].

Table 4 presents examples of droplet shapes obtained from contact angle measurements for water and diiodomethane. The table also gives the values of the contact angle θ, which is an indicator of wetting or spreading. For all applied slide-burnishing conditions and both measuring liquids, the contact angle Θ < 90° was obtained, which means that the measuring liquid is not spread over the surface but is wetted. This means that the liquid has the ability to maintain contact with the surface of the solid body, which may be an important feature in terms of interaction between two elements in the friction pair in the presence of the machining liquid. The largest contact angle was obtained for the surface slide burnished using emulsion where the measuring liquid was water, and for the surface after grinding where diiodomethane was used as a measuring liquid. It should be noted that there are differences in the shape and height of liquid drops. A drop of water is characterized by significantly less contact with the tested surface witha significant height at the same time. On the other hand, a drop of diiodomethane is characterized by a large contact with a small height at the same time.

## 4. Conclusions and Summary

This study was conducted on C45 unalloyed steel samples, which were slide-burnished with the use of various machining liquids. The following conclusions summarize the results of this study:

1. The effects of slide burnishing depend on the type of medium in which the process is carried out. As a result of the employed treatment, all studied properties of the surface layer changed.
2. After slide burnishing of elements made of C45 unalloyed steel using different machining fluids, the surface roughness parameters Ra and Rt decreased as expected. The surface roughness parameter Ra decreased by more than eight times, and Rt by more than five times compared to the value after grinding. Regarding the Abbott–Firestone curve parameters, the effect of slide burnishing was more complex. The reduced peak height decreased from 79% to 82%, while the depth of the surface roughness core decreased from 84% to 88% in relation to the value after grinding. Favourable changes can contribute to improving the functionality of these surfaces.
3. An unfavourable phenomenon observed after slide burnishing was a decrease in the Rvk parameter—its value reduced from 45% to 60%, which indicates a probable decrease in the retention capacity of the lubricant in the mating friction pair.
4. After slide burnishing, the 3D parameters of the surface roughness also changed: the topography changed (micro-irregularities were more deformed and irregular), the angle of inclination of the Abbott–Firestone curve also changed (the curve was more flattened), and the material ratio of Smr increased for the cut-off levels of c = 40% and c = 60%.The increase in the material ratio allows us to conclude that the materials are resistant to abrasive wear.
5. In terms of abrasive wear resistance, the greatest changes in 2D and 3D surface roughness, surface topography, and Abbott–Firestone curve were obtained when the slide-burnishing process was conducted using oil+PMM+MoS_2_ as a machining liquid.
6. The addition of PMM and MoS_2_ to the oil caused an about 25% increase in microhardness compared to the ground sample. The changes in microhardness after slide burnishing for the “richest” machining centre reached up to 20 μm.
7. After slide burnishing, similar values of surface free energy or a slight increase in the surface free energy were obtained, when compared to the values after grinding.

Based on the results of selected properties of the surface layer of C45 steel obtained after slide burnishing conducted with the use of various machining fluids, it can be concluded that the surface after slide burnishing with oil+PMM+MoS_2_ should meet the requirements for mating elements in a friction pair.

## Figures and Tables

**Table 1:** Technological parameters of slide burnishing.

No.	*F*, N	*f*, mm/rev.	*v*, m/min	Types of Liquid
1	180	0.04	35	oil with polymethyl methacrylate—PMM (0.5%) and molybdenum disulphide—MoS_2_ (0.5%) solutions
2				oil with a polymethyl methacrylate solution—PMM (0.5%)
3				oil with molybdenum disulphide—MoS_2_(0.5%)
4				Mobile Vactra^TM^ Oil No. 2
5				EcoEm-1 emulsion

**Table 2:** Surface topography of C45 steel after grinding (pre-treatment) and slide burnishing with the use of various cutting fluids (*F* = 180 N, *f* = 0.04 mm/rev., *v* = 35 m/min).

**Pre-Treatment**	**oil+PMM+MoS_2_**
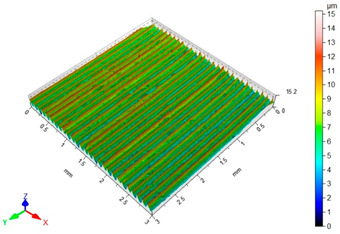	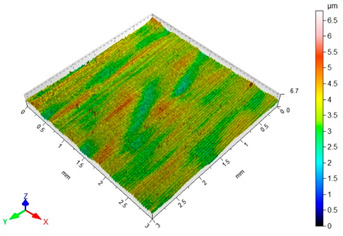
Sa = 1.70 μm, Sz = 15.2 μm, Sp = 8.95 μm, Sv = 6.28 μm, Sku = 2.65, Ssk = 0.564	Sa = 0.40 μm, Sz = 6.80 μm, Sp = 3.26 μm, Sv = 3.54 μm, Sku = 3.05, Ssk = −0.076
**oil+PMM**	**oil+MoS_2_**
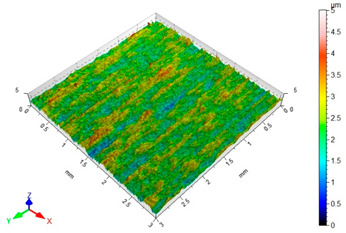	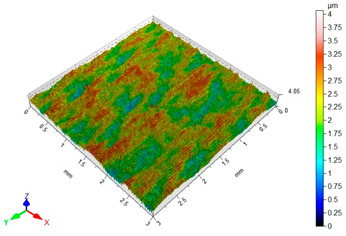
Sa = 0.38 μm, Sz = 5.01 μm, Sp = 2.81 μm, Sv = 2.21 μm, Sku = 3.27, Ssk = −0.059	Sa = 0.39 μm, Sz = 4.07 μm, Sp = 2.02 μm, Sv = 2.05 μm, Sku = 2.80, Ssk = −0.055
**oil**	**EcoEm-1 emulsion**
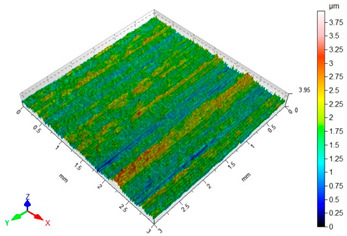	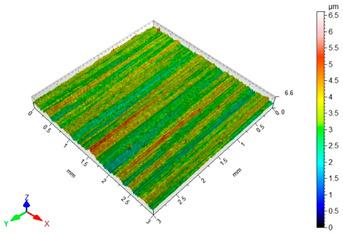
Sa = 0.33 μm, Sz = 3.96 μm, Sp = 2.37 μm, Sv = 1.58 μm, Sku = 3.07, Ssk = 0.090	Sa = 0.43 μm, Sz = 6.61 μm, Sp = 3.40 μm, Sv = 3.22 μm, Sku = 3.45, Ssk = 0.173

**Table 3:** Material bearing curve for C45 steel after grinding (pre-treatment) and slide burnishing conducted with various cutting fluids (*F* = 180 N, *f* = 0.04 mm/rev., *v* = 35 m/min).

**Pre-Treatment**	**oil+PMM+MoS_2_**
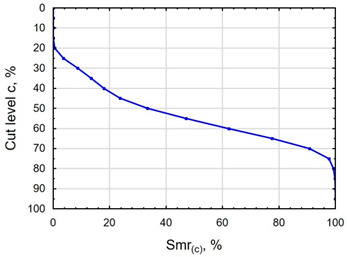	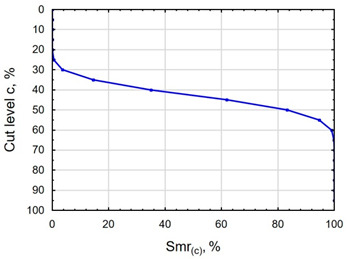
**oil+PMM**	**oil+MoS_2_**
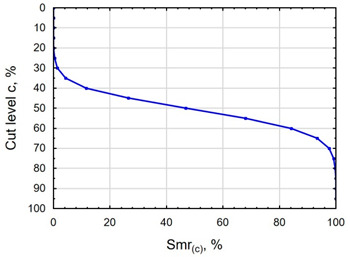	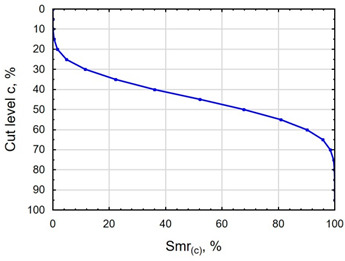
**oil**	**EcoEm-1 emulsion**
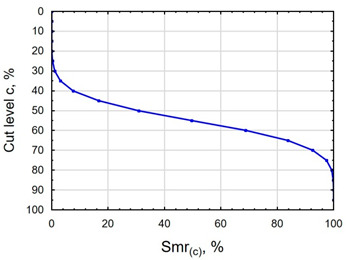	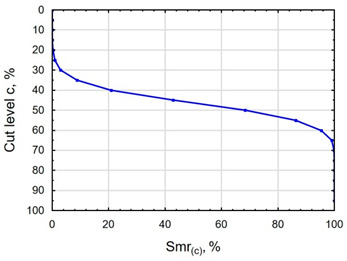

**Table 4:** Photographs of measuring liquid drops (volume—4 μL) used in contact angle measurements (where: 1—slide burnishing in oil+PMM+MoS_2_, 2—slide burnishing in oil+PMM, 3—slide burnishing in oil+MoS_2_, 4—slide burnishing in oil, and 5—slide burnishing in EcoEm-1 emulsion).

Type of Liquid	Measuring Liquid	
	Distilled Water	Diiodomethane
1	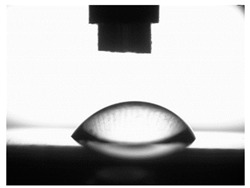	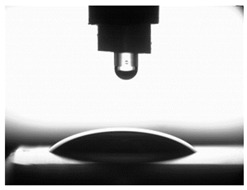
	Θ = 66.69° ± 1.51°	Θ = 32.03° ± 1.78°
2	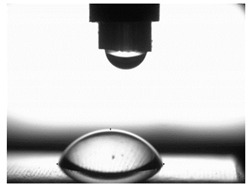	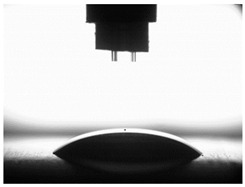
	Θ = 69.75° ± 2.01°	Θ = 31.91° ± 1.71°
3	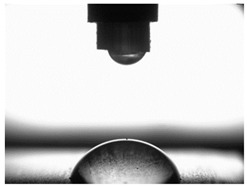	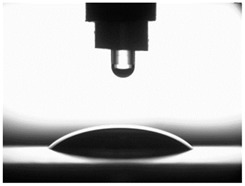
	Θ = 70.69° ± 1.56°	Θ = 33.89° ± 2.29°
4	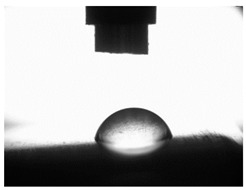	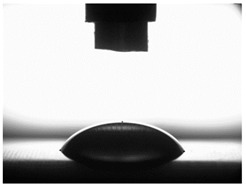
	Θ = 71.56° ± 1.67°	Θ = 34.35° ± 1.13°
5	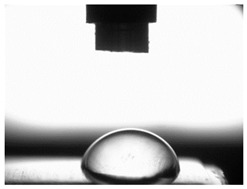	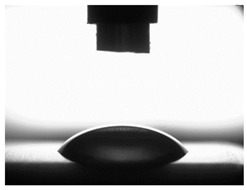
	Θ = 75.67° ± 1.73°	Θ = 29.68° ± 1.50°
6	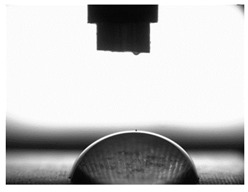	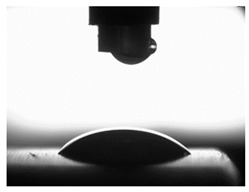
	Θ = 66.37° ± 3.85°	Θ = 36.68° ± 0.79°

## Data Availability

Not applicable.
